# Use of the Patient Health Questionnaire (PHQ-9) in Practice: Interactions between patients and physicians

**DOI:** 10.1177/1049732320924625

**Published:** 2020-06-20

**Authors:** Joseph Ford, Felicity Thomas, Richard Byng, Rose McCabe

**Affiliations:** 1 University of Exeter, Exeter, United Kingdom; 2 University of Plymouth, Plymouth, United Kingdom; 3 City, University of London, London, United Kingdom

**Keywords:** mental health and illness, primary, health care, depression, mental health and illness, qualitative, conversation analysis, United Kingdom

## Abstract

We analyze the use of nine-item Patient Health Questionnaire (PHQ-9), an instrument that is widely used in diagnosing and determining the severity of depression. Using conversation analysis, we show how the doctor deploys the PHQ-9 in response to the patient’s doubts about whether she is depressed. Rather than relaying the PHQ-9 verbatim, the doctor deviates from the wording so that the response options are selectively offered to upgrade the severity of the patient’s symptoms. This works in favor of a positive diagnosis and is used to justify a treatment recommendation that the patient previously resisted. This contrasted with the rest of the data set, where diagnosis was either not delivered (as patients are presenting with ongoing problems) or delivered without using the PHQ-9. When clinician-administered, the PHQ-9 can be influenced by how response items are presented. This can lead to either downgrading or upgrading the severity of depression.

## Introduction

**Figure 1.**
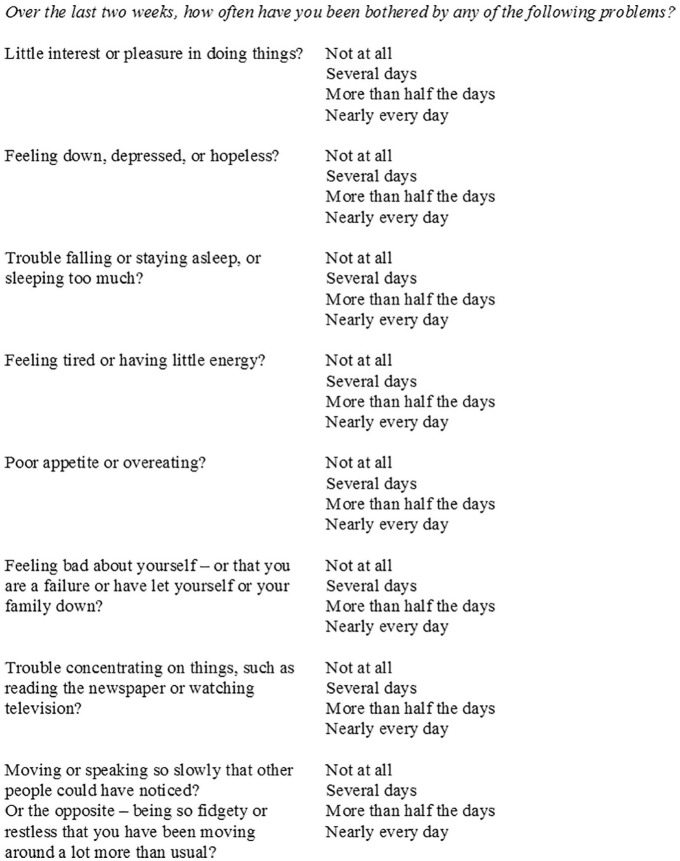
Nine-item Patient Health Questionnaire (PHQ-9).

Diagnosing and determining the severity of depression is seen as an important element of practice in primary care. Multiple scales and questionnaires have, as a result, been developed to this end, one of which is the nine-item Patient Health Questionnaire (PHQ-9; Kroenke et al., 2001). A patient self-report measure (although one that can also be administered by doctors), the PHQ-9 was developed with the aim of providing a questionnaire that combined brevity with “construct and criterion validity” (Kroenke et al., 2001, p. 612). The PHQ-9 asks patients to rate, on a four-point scale ranging from “not at all” to “most days,” the frequency with which they have experienced certain depression symptoms in the preceding 2 weeks (see Figure 1). Researchers have, in the years since its development, confirmed the PHQ-9’s validity and reliability in various contexts (Cameron et al., 2008; Lowe et al., 2004; Martin et al., 2006; Titov et al., 2010).

However, as Malpass et al. (2016) note, this psychometric credibility does not necessarily accord with the lived experience of being depressed as explored in qualitative analysis. Although patients in interview studies generally agree, for example, that having a numerical indication of their depression severity can be useful and validating (Dowrick et al., 2009; Malpass et al., 2016), they also suggest that the PHQ-9 does not necessarily reflect their lived experiences of depression (Malpass et al., 2010, 2016). Following the introduction of the use of questionnaires to measure severity of depression as a part of the quality Outcomes Framework financial incentives scheme, general practitioners (GPs) have also described mixed views. Some appreciate the opportunity to delegate the diagnostic process to a putatively “objective” tool (Tavabie & Tavabie, 2009); other GPs, though, are skeptical of depression severity questionnaires and doubt their validity and utility (Dowrick et al., 2009). Although the scores on the PHQ-9 have been graded according to different levels of severity of depression, it is not designed as a diagnostic tool. In addition to initially being used to measure outcomes in U.K. primary care, it has been used as an outcome measure in trials, as a means of determining who should be included in studies, as a means of case identification, and to monitor the effectiveness of new systems of care.

There is little research on how such diagnostic questionnaires are used in practice. In other contexts, there is a body of work on how questionnaires are introduced, deployed, and influenced by what is happening in the given interaction, that is, the interactional context. Most prior research on the interactional context of standardized instruments has focused on survey interviews. The most extensive study is the one by Houtkoop-Steenstra (2000), who shows two contrasting ways in which interactional context is relevant. On one hand, an interviewer can deviate from the strictures of the instrument, administering it in a conversational manner. While defenders of standardized tools might argue that this simply marks a deviation from a neutral ideal, Houtkoop-Steenstra (2000) shows that rigid adherence to a predetermined procedure can also be interactionally problematic by, for example, leading the interviewer to ask for information that the participant has already given.

Houtkoop-Steenstra’s (2000) findings are echoed by a range of studies showing how both interviewer and participant deviate from the neutral formats of a survey interview (de Vries et al., 2014; Grindsted, 2005; Maynard & Schaeffer, 2006; Suchman & Jordan, 1990). They are also supported by a small but significant body of similar research in other environments. Martinell Barfoed (2018), for example, shows how social workers using the Addiction Severity Index can “soften” difficult, delicate, or awkward questions by stepping outside of the standardized frame and adding a “meta-comment” (e.g., “These are tricky questions!” (p. 45)).

Jones et al. (2019) analyze the use of Adenbrooke’s Cognitive Examination (ACE III) in a memory clinic. Specifically, they show how clinicians differ in their delivery of this standardized test by, for example, asking questions in a variety of ways (the ACE III provides guidance but not a verbatim script for clinicians to follow) and offering additional help to patients who are struggling to answer. As they note, there is a “key tension” between the standardization of the test and the different approaches taken by practitioners, which “adds an interactionally unique dimension” (Jones et al., 2019, p. 9).

Antaki (1999), meanwhile, analyzes standardized quality-of-life interviews with individuals with intellectual disabilities. He shows how interviewers edit the wording on the questionnaire in a way that lowers the criteria needed to get a high score. This form of deviation from the official wording could, he notes, lead to inflated scores that provide an inaccurate picture of respondent’s lives—an outcome that might be “against [their] best interests” (p. 451) (see also Heritage et al., 2007, on how seemingly small variations in language can impact the outcome of a medical interaction).

However, there has, to our knowledge, been no published research on the in situ use of depression diagnostic questionnaires. The objective of this study, therefore, is to use recorded primary care consultations to explore (a) the diagnostic use of the PHQ-9 in practice and (b) how this contrasts with diagnosis without the PHQ-9 and cases where there is no diagnosis.

## Method

### Data

This study was developed as part of the DeStress Project on mental health in low-income communities (Thomas et al., 2019). The consultations analyzed are part of a wider corpus of 52 video- and audio-recorded GP–patient mental health consultations taken from the One in a Million archive. This is an archive of 300 primary care consultations collected in the west of England from 2014 to 2015 (Barnes, 2017).^7^

Some of the 52 recordings were consultations where the patient was presenting with mental health problems alone (*n* = 21), whereas in others the patient was presenting with both physical and mental health problems (*n* = 31). Most patients had some prior history of mental health problems, although they were at varying points in the treatment process: Some had come to the GP seeking treatment, whereas others were already taking treatment and were visiting their GP for a follow-up.

Ethical permission and informed consent were obtained as part of the original data collection for the One in a Million study (Jepson et al., 2017). Ethical permission for the use of these recordings in the DeStress Project was granted by the Cambridgeshire and Hertfordshire National Health Service (NHS) Research Ethics Committee. Once transferred, the recordings and associated patient data were stored on a secure university drive and were accessible only by members of the research team.

### Data Analysis

We analyzed all consultations to identify instances of diagnosis delivery. We identified only two cases of diagnosis delivery because patients are mostly presenting with ongoing problems: In these cases, a diagnosis would be not expected. As diagnosis typically precedes treatment discussions, we show how treatment discussions typically occur in these cases where a diagnosis is not present. We then focus on the two cases where a diagnosis is delivered: one case without the PHQ-9 questionnaire and one with the PHQ-9 questionnaire.

The relevant parts of the diagnostic sequences were transcribed in detail using the Jeffersonian (Jefferson, 2004) conventions (see Supplemental Material for a glossary) and analyzed using conversation analysis (CA). CA is a micro-analytic approach that focuses on what speakers say (e.g., their lexical choice), how they say it (e.g., their intonation or nonverbal behavior), and the point at which they say it. Applied to medical interaction, it has been used to study, among other things, how patient concerns are elicited (Heritage et al., 2007), how the subtle wording of recommendations for treatment displays different opportunities for patient involvement in decision-making about starting treatment (Stivers et al., 2018), and how diagnoses are delivered (Peräkylä, 1998).

## Results

First, we describe consultations where there is no diagnosis or diagnosis is present without the PHQ-9. Second, we describe how the PHQ-9 is used to establish a diagnosis.

### Standard Consultations

Our data set can be divided into three groups: consultations in which no diagnosis is present (*n* = 50), a consultation in which a diagnosis is present prior to treatment discussion (*n* = 1), and the case where the doctor uses the PHQ-9 to diagnose the patient with depression (*n* = 1).

The first category encompasses a range of consultations. In some of these, one might not expect a diagnosis to be present (e.g., follow-up consultations reviewing an ongoing problem and/or renewing a prescription). In others, though, one could say that diagnosis is noticeably absent. This is because they involve the doctor recommending that the patient initiate a treatment, and such recommendations typically directly follow diagnosis in the primary care consultation (Robinson, 2003).

**Figure 2.**
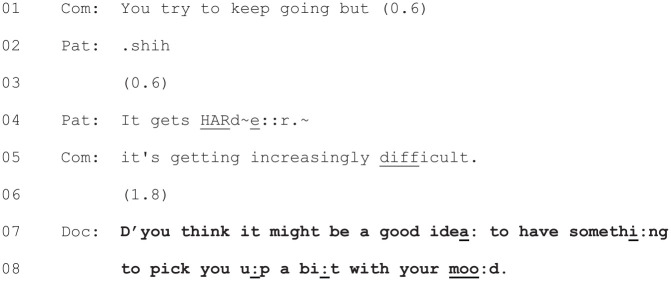
[28: 14.13/21.38].

This is not the case for most of the data set, however, largely because most of the consultations involved follow-up rather than new visits. An illustration of this can be seen in Figure 2, which begins as the patient and her partner are describing how her physical health has impacted her mood. The doctor’s treatment recommendation is marked in boldface.

The doctor’s treatment recommendation here can be seen at Lines 7–8, where she raises the possibility of the patient having “something to pick [her] up a bit with [her] mood.” However, in a deviation from Robinson’s (2003) model, this move to the treatment phase of the consultation is not preceded by a diagnosis of the patient’s condition. Nor is there a diagnosis present elsewhere in the consultation. This example is broadly representative of most of the consultations in the data set where a new treatment is started. Robinson’s (2003) model of diagnosis followed by treatment discussion was based on patients presenting with acute illnesses for the first time in primary care. In the current data, most of the patients have a history of mental health problems and/or mental health treatment. In Figure 2, for example, the patient’s partner’s utterance at Line 5 (“it’s getting *increasingly* difficult”) makes it clear that this is not a new problem.

**Figure 3.**
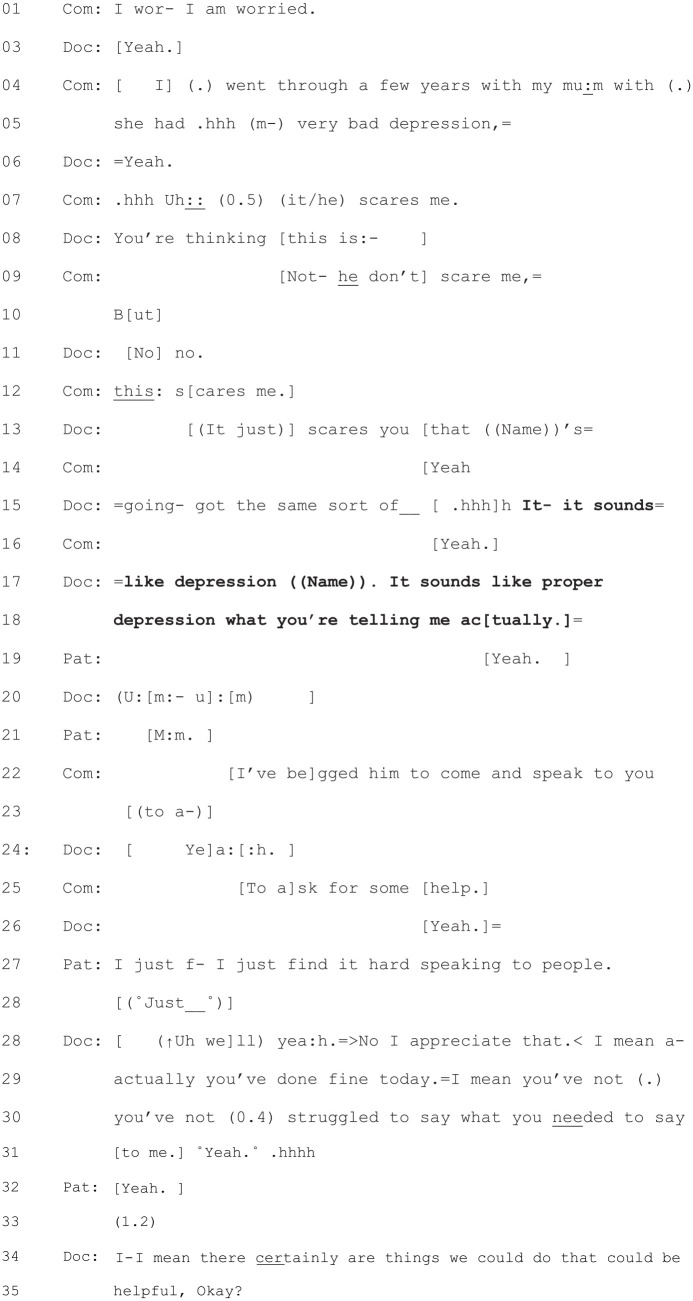
[44: 9.09/17.22].

Fifty of the consultations in the data set do not feature a diagnosis. This leaves two consultations in which there was a diagnosis. The first of these can be seen in Figure 3.

This diagnosis at Lines 15 and 17–18 follows the patient’s (and his wife’s) problem presentation and the patient’s wife’s candidate diagnosis of depression (Line 5). This is accepted by the patient (yeah) in Line 19. Following intervening talk about how the patient’s wife had entreated the patient to come and the patient’s reluctance to come, the diagnosis is followed by the doctor’s initiation of treatment discussion at Lines 34–35. Earlier in this consultation, the doctor has made an explicit reference to this being a new problem for the patient (“It’s not something we normally talk about, is it?”), hence the diagnosis: “It sounds like depression.”

### Diagnosis Using the PHQ-9

The case we now focus on features a diagnosis after treatment discussion, rather than a diagnosis followed by treatment discussion. The GP in this consultation is a man in his 50s who has worked at the practice for more than 10 years. The patient is a woman in her 70s suffering from a variety of problems, both physical (vertigo and breathing problems) and psychosocial (money problems and a feeling of social isolation). The patient was previously taking Valium to help with feelings of anxiety.

**Figure 4.**
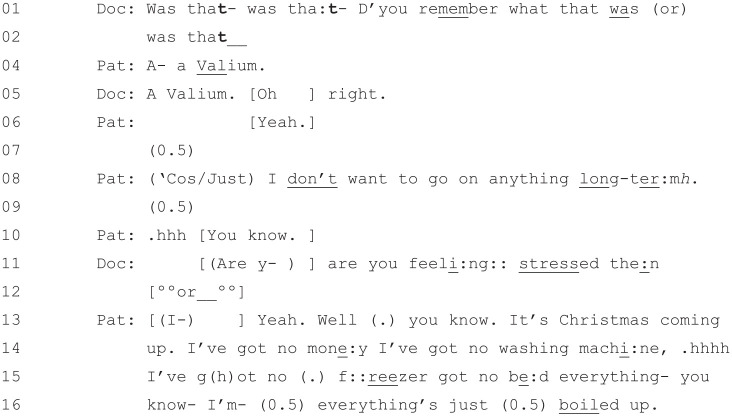
[36: 01.02/25.51].

An extract of the patient describing her psychosocial problems, taken from about a minute into the consultation, can be seen in Figure 4.

While short, this extract captures the focus of the first 8 minutes of the consultation, with the patient describing the various issues with which she is struggling. With the stress of Christmas coming up, she indicates at Line 8 that she would like some more Valium rather than anything long-term (i.e., antidepressants).^1^ After around 8 minutes, the doctor suggests that they focus on the patient’s breathing. The patient agrees, and the next 10 minutes of the consultation are focused exclusively on physical health issues.

### How the PHQ-9 Is Interactionally Occasioned

**Figure 5.**
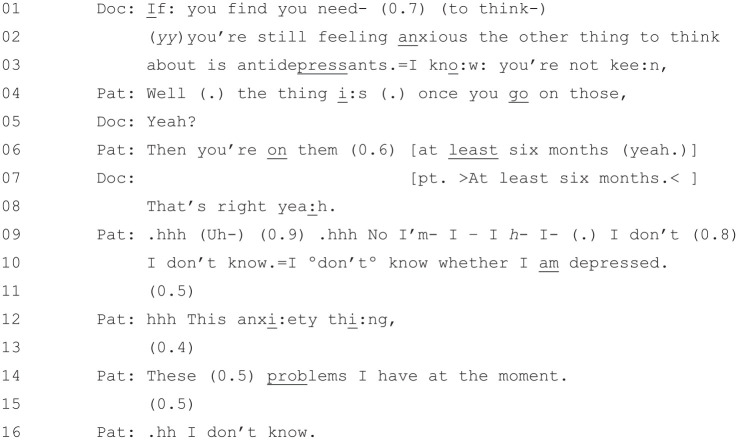
[36: 17.48/25.51].

Around 18 minutes into the consultation, the doctor and patient wrap up discussion about physical health matters and the doctor brings the discussion back to mental health. The moment at which he does so can be seen at the start of Figure 5

The doctor starts this extract at Lines 1–3 by suggesting that the patient might “think about . . . antidepressants” if she is “still feeling anxious.” Notably, the doctor also acknowledges the patient’s aforementioned reluctance expressed in Figure 4: “=I kno:w you’re not kee:n,”. Note also that the doctor has transitioned into treatment discussion without issuing a diagnosis, in line with the pattern in Figure 2 (label the cases?).

The patient, from Line 4 onward, actively resists (Heritage & Sefi, 1992; Stivers, 2005) the doctor’s suggestion.^2^ First, at Lines 4 and 6, she expresses concern about the amount of time that she would be on them: “Well (.) the thing i:s (.) once you go on those, . . . Then you’re on them (0.6) at least six months (yeah.)” (the “well” preface here indicates that the patient’s response will not be aligned with the doctor’s recommendation; Heritage, 2015). After the doctor has confirmed the likely duration of the treatment, the patient, from Line 9 onward, offers a different reason for not wanting to take antidepressants: that she is not “sure if [she is] depressed.”

**Figure 6.**
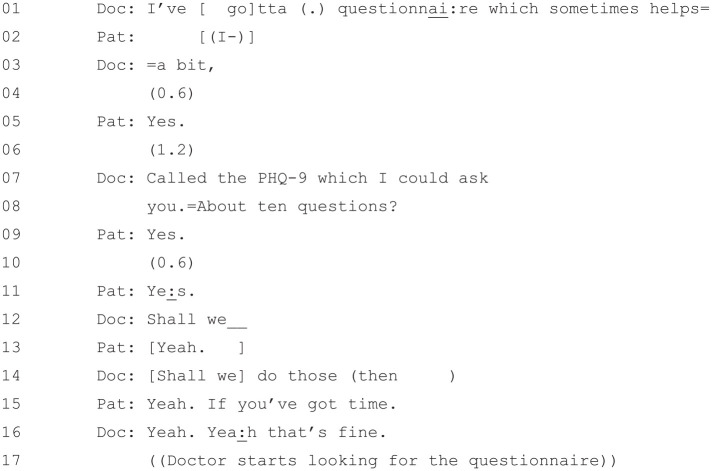
[36: 18.21/25.51].

It has been shown that when patients resist treatment recommendations, it is incumbent upon the doctor to address their resistance before the consultation can proceed (Stivers, 2005). It is at this point, which follows directly on from Figure 5, that the doctor suggests using the PHQ-9 questionnaire (Figure 6).

The doctor, at Lines 1, 3, and 7–8, suggests using a questionnaire to help. This is seemingly in line with Stivers (2005), who notes that doctors will frequently “retreat to previous activities” such as “restating diagnostic findings” (p. 49) when faced with patient resistance. However, Stivers’s (2005) wording assumes, as was discussed in the previous section, that “the activity of treatment is contingent upon that of diagnosis” (Robinson, 2003, p. 31). However, there has been no diagnosis earlier in the consultation for the doctor to “retreat to”—rather, he is invoking diagnosis in *response to* the patient’s doubt about whether she is depressed that has arisen during the treatment phase. The PHQ-9 is then used to provide an evidential basis for a diagnosis of depression. Both diagnosis and the tool used to accomplish it (i.e., the PHQ-9), then, have been occasioned by a local interactional reason, that is, the need to get buy-in from the patient on the proposed treatment.

### How the PHQ-9 Is Interactionally Administered

**Figure 7.**
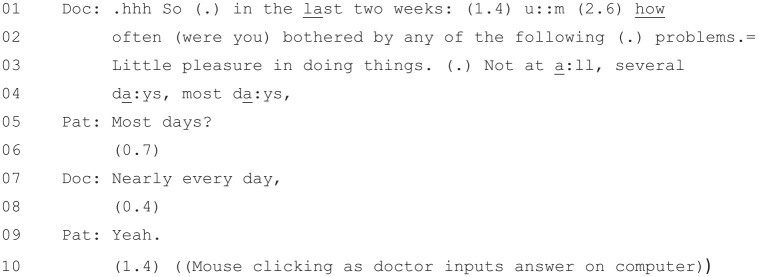
[36: 19.10/25.51].

Figure 7 begins as the doctor is reading out the first item on the PHQ-9.

The doctor’s reading of the beginning of the questionnaire from Lines 1–4 and the first item at Line 3 (“Little pleasure in doing things”) is virtually verbatim. However, as he reads out the response *options*, he begins diverging from the written version. While he reads out verbatim the first two low-scoring answers from Lines 3–4 (“Not at a:ll, several da:ys”), for example, at Line 4 he offers an answer that is not on the questionnaire: “most da:ys,”. This appears to be a gloss of the final two high-scoring responses (“More than half the days” and “Nearly every day”), and the patient responds affirmatively to it at Line 5. The doctor then offers only the higher scoring of the two official answers at Line 7 (“Nearly every day,”), and the patient again, at Line 9, answers in the affirmative.

**Figure 8.**
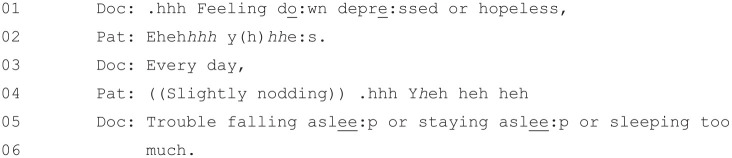
[36: 19.29/25.51].

Based on the first item alone, we can see that the doctor is not simply acting as a “relayer” (Houtkoop-Steenstra, 2000) for the PHQ-9, reading out word for word what it says. Instead, he is modifying the questionnaire as he goes, particularly when it comes to the possible responses that the patient can give to each item. This may also be based on the patient’s previous account in the consultation about her situation. This is a pattern which continues as the doctor moves onto the second item (Figure 8).

This extract begins similarly to Extract 5, with the doctor reading Item 2 verbatim at Line 1: “Feeling do:wn depre:ssed or hopeless,”. However, rather than providing the full list of possible response options, he instead leaves a space for the patient to respond at Line 2. This response (an aspirated and laughter-inflected “yes”) is not a response option on the questionnaire but indicates that the “not at all” option would not be relevant. Rather than reading out verbatim the three remaining possible responses, though, the doctor again offers only one possible response (“Every day,”) that is not present on the questionnaire and suggests a severity beyond that indicated by any of the official responses (the most severe of which is “*Nearly* every day”).^3^ The patient once again confirms at Line 4.

The preceding two extracts are indicative of how the rest of the PHQ-9 is administered, with the doctor reading almost verbatim the *items* of the questionnaire but being selective in offering possible *responses to* those items. These selective readings seem to favor a positive diagnosis of depression, as the options he is offering are typically from the higher frequency end of the spectrum. Moreover, he is not doing this in a vacuum but is building upon the patient’s own spontaneous responses to his reading of the items, which themselves favor a positive response.

**Figure 9.**
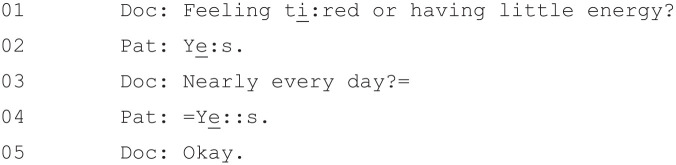
[36: 19.50/25.51].

Occasionally, the doctor’s loose reading is merely selective. Consider, for example, his reading of the fourth item (Figure 9).

In this case, the doctor does, at Line 3, read out verbatim one of the official responses on the PHQ-9: “Nearly every day?” Again, though, this is only the highest scoring of three possible responses (assuming that the patient’s affirmative response at Line 2 rules out “not at all”), the other two of which would contribute less to the patient’s overall score. This can be attributed, again, to the information that the patient has already given about her state of mind.

**Figure 10.**
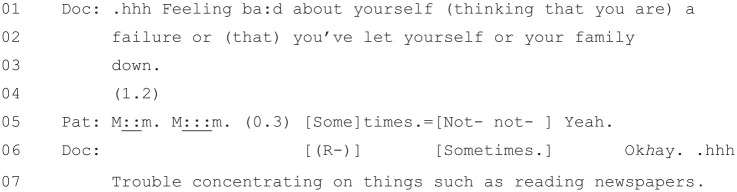
[36: 20.02/25.51].

In some cases, it is the patient who offers responses that are not officially part of the questionnaire, as can be seen in the doctor’s reading of the sixth item (Figure 10).

At Line 5, after some hesitation, the patient offers a mildly affirmative response to the doctor’s reading of the item: “M::m. M:::m. (0.3). Sometimes.” “Sometimes” is not an official answer on the PHQ-9, yet the doctor does not attempt to clarify or translate it into the terms of the questionnaire (cf. Houtkoop-Steenstra, 2000). Instead, he offers a confirmation which closes down this item at Line 6 (“Ok*h*ay.”) before moving onto the next item at Lines 6–7.

To summarize, the doctor here has taken the patient’s statements about her condition and fitted them to the symptom categories in the PHQ-9. In doing so, however, he has not acted simply as a neutral “relayer” (Houtkoop-Steenstra, 2000; see also Clayman and Heritage, 2002, on “neutralism”) for the text. Instead, he has given the patient response options that are (a) not necessarily on the official questionnaire, (b) responsive to the patient’s utterances, and (c) slanted toward a positive diagnosis of depression.^4^

The analysis shows how, in this primary care consultation, the response items on the PHQ-9 are modified and the responses steered toward higher severity responses. This has been responsive to the patient, who has herself introduced interactional elements into her responses (e.g., her accompanying laughter in Figure 8). This leads to a positive diagnosis of depression used in support of recommending antidepressants, which may be seen in the next extract.

### The Outcome of the PHQ-9

**Figure 11.**
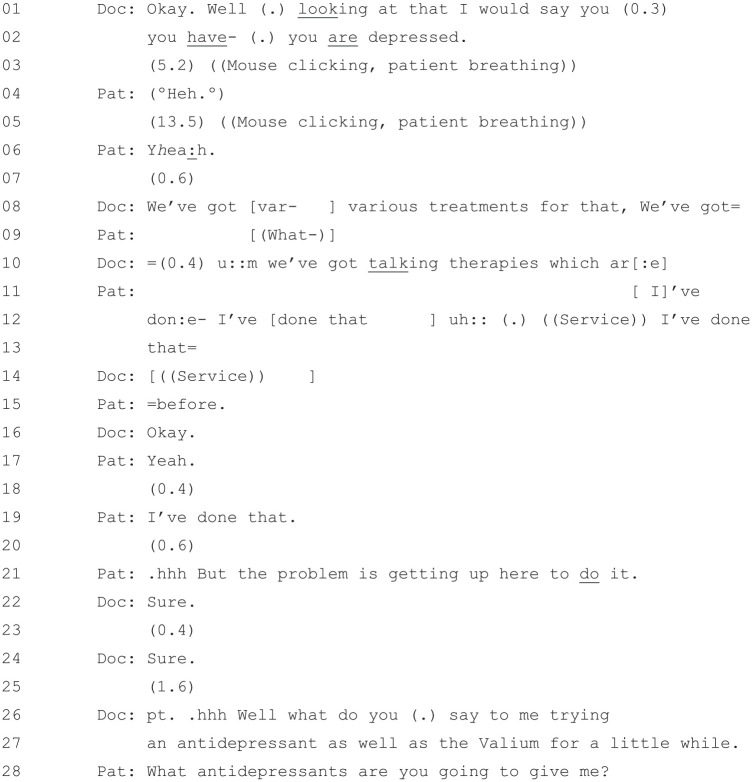
[36: 21.19/25.51].

Figure 11 starts directly after the doctor has finished reading through the PHQ-9 with the patient.

The doctor offers his diagnosis based on the PHQ-9 at Line 1: “Well (.) looking at that I would say that you (0.3) you have- (.) you are depressed.” Peräkylä (1998) notes three types of diagnostic statements: those which plainly assert the diagnosis (e.g., “There’s still an infection”), those which offer an inexplicit reference to evidence (e.g., “There appears to be (1.0) an infection”), and those which offer an explicit reference to the evidence (e.g., describing at length specific symptoms). The doctor’s statement in this extract seems closest to the third type, alluding to the specific diagnostic tool that he has used to establish his diagnosis and, by implication, the symptoms that the patient has described when going through it.

The doctor’s diagnostic statement is followed by two long silences, interspersed with minimal responses from the patient at Lines 4 (“Heh”) and 6 (“Y*h*ea:h”). Such minimal responses are typical when it comes to diagnoses which, unlike treatment recommendations, do not call for patient endorsement before the consultation can proceed (Heath, 1992). Given this, we might ask why the doctor went to the trouble of going through the PHQ-9 when he could, seemingly, have just offered a “plain assertion” (Peräkylä, 1998) of his own opinion. It is worth noting, in answer to this, that doctors in Peräkylä’s (1998) analysis overwhelmingly made clear the evidential basis of their diagnostic claims—even “plain assertions,” by being positioned directly after (or during) physical examination, performed this function. The PHQ-9 in this context performs, to some extent, the same kind of function that an examination performs in a physical health context, fitting the patient’s subjective descriptions to a set of symptom categories that can, in turn, be cited as the basis for a diagnosis (see also Dowrick et al., 2009).

After the patient’s acceptance of the diagnosis, and after an extended period of silence, the doctor does indeed proceed to recommend treatment. He first offers “talking therapies” at Line 10, but the patient in Line 11 states that she has “done that before.” (Lines 12–14) but had trouble “getting up here to do it.” (Line 20). The talking therapy option is thus abandoned, and the doctor returns, at Lines 25–26, to offer the same treatment that he offered in Figure 4: “Well what do you (.) say to me trying an antidepressant as well as the Valium for a little while.”. The doctor’s return to his original activity is again in line with Stivers (2007), who notes that, after a doctor has “retreat[ed] to a previous [activity]” in response to patient resistance, they will typically “then proceed again through the remaining activity phases back to treatment recommendation” (p. 111).

**Figure 12.**
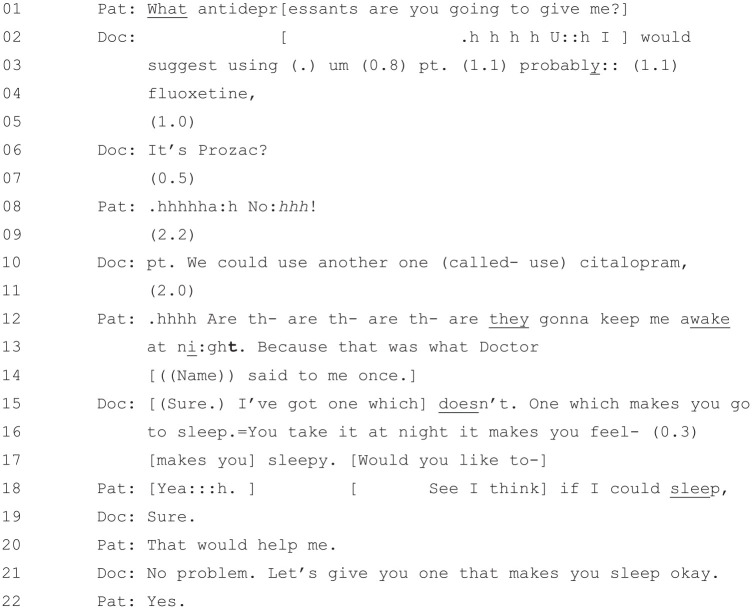
[36: 22.07/25.51].

The patient’s response to the reiterated treatment recommendation at Line 27 is to ask the doctor “what antidepressants” he is “going to give [her]?” This response is significant because it shows that, in contrast to her earlier expressed reluctance, the patient is not resisting taking an antidepressant as strongly as she did previously.^5^ In the following extract (Figure 12), the patient has not yet accepted taking an antidepressant and queries which type of antidepressant the doctor is going to give her.

The doctor’s first suggestion in this extract is that the patient try “fluoxetine” (Line 4) or, as he clarifies at Line 6, “Prozac.” The patient responds very negatively to this suggestion at Line 8 (“.hhhhha:h No:*hhh*!”^6^), and after further discussion, they agree at Lines 21–22 that the patient will try a different antidepressant that will help her to sleep. Although there is still negotiation about what antidepressant will be prescribed, the patient has gone from strongly resisting an antidepressant, to negotiating about an antidepressant, to ultimately agreeing to take an antidepressant that is sedative—a common form of turnaround in this context (Ford et al., 2019). A key to the turnaround in this case has been the doctor using the PHQ-9 to provide an “evidential basis” (Peräkylä, 1998) to support his treatment recommendation. This is in line with Heritage and McArthur (2019), who note that diagnoses that occur after treatment recommendations (as opposed to the standard, pretreatment recommendation position noted above) “often serve as embedded or post hoc justifications for those recommendations” (p. 266).

## Discussion

The objective of this study was to explore (a) the diagnostic use of the PHQ-9 in primary care and (b) how this contrasts with diagnosis without the PHQ-9 and cases where there is no diagnosis. We found that the PHQ-9 was deployed in response to the patient’s expressed resistance to treatment, which was itself grounded in her uncertainty about whether she was depressed. The way in which the PHQ-9 was administered was not neutral. Rather, the way that the response options were offered and the patient’s answers interpreted were influenced both by the person administering the questionnaire and by the patient’s initial verbal and nonverbal responses. The response options offered were on some occasions modified to present response options not in the questionnaire and/or were presented to upgrade the severity of the patient’s symptoms. This provided evidence for a diagnosis of depression which, in turn, was used to support a recommendation for antidepressants (cf. Heritage & McArthur, 2019), ultimately overcoming the patient’s earlier resistance to this form of treatment.

This is, to our knowledge, the first published study on the in situ use of the PHQ-9 questionnaire in a mental health context. Although the doctor’s use of the PHQ-9 is not suggested as typical, it is clear that how it was used influenced the diagnostic outcome. Specifically, we have shown how the doctor’s presentation of response options was modified and/or slanted in favor of upgrading the severity of the patient’s symptoms and generating a higher score. This finding has important implications for practitioners who use the PHQ-9 and other such questionnaires, highlighting how subtle ways in which they administer an instrument affect the diagnostic outcome.

Before analyzing the case featuring the PHQ-9, we considered both cases where there was no diagnosis (Robinson, 2003) and a case with diagnosis in the expected slot. The PHQ-9 case sits outside both of these categories. The PHQ-9 and the resultant diagnosis came about only because the patient questioned a diagnosis of depression by resisting the doctor’s recommendation for antidepressants (“I don’t know if I am depressed”). Had she not expressed this resistance, there is no reason to believe that the consultation would not have unfolded as it did in the other examples, that is, without any diagnosis. Our analysis shows, therefore, that diagnosis can in fact be used to do things other than diagnosing—in this case, to address a patient’s treatment resistance.

This article contributes to the existing body of qualitative research on mental health questionnaires (Dowrick et al., 2009; Malpass et al., 2010, 2016; Tavabie & Tavabie, 2009) and, more broadly, on how standardized instruments are used in interaction (de Vries et al., 2014; Grindsted, 2005; Houtkoop-Steenstra, 2000; Jones et al., 2019; Maynard & Schaeffer, 2006; Suchman & Jordan, 1990). Our findings especially parallel those of Antaki (1999), who showed how wording of items on a quality-of-life questionnaire could potentially lead to score inflation in interviews with individuals with intellectual disabilities. However, we would note some important differences. First, in Antaki (1999), the interviewers typically edited the questions; here, on the contrary, the doctor deviated from the PHQ-9 largely in his delivery of the *response options* to the questions.

There are also differences in the motivation for the editing and outcome of such questionnaires. Antaki (1999) suggests that quality-of-life interviewers might have edited the questions in a way that would make it easier, both cognitively and socially, for the respondents to answer them. In this consultation, however, the doctor’s rewording of the PHQ-9 was done in a way that was responsive to the patient’s own responses. Moreover, whereas the quality-of-life scores in Antaki’s (1999) study were to be entered into respondents’ official records (where they could influence key decisions about the support that they were to receive), the outcome of the PHQ-9 in this consultation fulfilled a more local function: to provide an “evidential basis” (Peräkylä, 1998) for a diagnosis of depression that could, in turn, support the doctor’s recommendation for antidepressants.

Our in-depth analysis has shown how subtle differences in how the response options of the PHQ-9 are offered positively favor a diagnosis of depression. However, there were insufficient cases to consider other uses of the PHQ-9 and similar questionnaires. Further research could collect further consultations where the PHQ-9 and other diagnostic instruments are used and consider both the range of ways in which they are deployed and the impact that this has on the outcome when it is used to establish a diagnosis of depression.

In conclusion, the PHQ-9 is a widely used tool in primary care for diagnosing depression and determining depression severity. For practitioners, it can provide an appealing numerical and “objective” diagnosis (Tavabie & Tavabie, 2009). However, practitioners should be aware that when administered by clinicians, the PHQ-9 is likely to be influenced by the way in which the response items are presented, which in itself is influenced by the patient’s previous accounts and may lead either to downgrading or upgrading the severity of depression. The PHQ-9 can also, in practice, be intertwined with interactional tasks that go beyond mere diagnosis or severity measurement.

## Supplemental Material

sj-pdf-1-qhr-10.1177_1049732320924625 – Supplemental material for Use of the Patient Health Questionnaire (PHQ-9) in Practice: Interactions between patients and physicians Click here for additional data file. Supplemental material, sj-pdf-1-qhr-10.1177_1049732320924625 for Use of the Patient Health Questionnaire (PHQ-9) in Practice: Interactions between patients and physicians by Joseph Ford, Felicity Thomas, Richard Byng and Rose McCabe in Qualitative Health Research

## Notes

1. Although she has not used the term “antidepressants,” we infer that “long-term” is referring to them because (a) she has contrasted “long-term” with “Valium,” a short-term medicine for anxiety and depression, and (b) she has used the colloquial term “go on,” which applies to medication but not alternative forms of long-term treatment for anxiety and depression; one cannot “go on therapy,” for example.

2. Interestingly, both aspects of the patient’s resistance (fear of long-term use and a reluctance to see her illness as depression per se) echo negative attitudes to antidepressants among older patients found by Givens et al. (2006).

3. Houtkoop-Steenstra (2000) refers to such close, but not quite verbatim, versions of response items as “near-formatted.”

4. While we do not know the patient’s exact score, a lower estimate puts it above 20, which is beyond the “Severe depression” threshold.

5. Although her suggestion that antidepressants are something that the doctor is going to “give [her]” perhaps displays some awareness that the medication is the doctor’s project rather than her own idea.

6. The extremity of her response can perhaps be attributed to the popular associations of the trade name “Prozac” (see Montagne, 2001).

7. The data on which this paper is based did not involve the collection of new data. Anonymised interview and focus group transcripts from participants involved in the wider DeStress study who consented to data sharing, plus additional supporting information, are available from the UK Data Service, subject to registration, at: 10.5255/UKDA-SN-853788.
