# Supplementary material for: Use of the Patient Health Questionnaire (PHQ-9) in Practice: Interactions between patients and physicians
Source: Qual Health Res. 2020 Jun 20;30(13):2146–59. doi: 10.1177/1049732320924625 (PMC7549295; doi:10.1177/1049732320924625)
Supplement: sj-pdf-1-qhr-10.1177_1049732320924625 – Supplemental material for Use of the Patient Health Questionnaire (PHQ-9) in Practice: Interactions between patients and physicians [file sj-pdf-1-qhr-10.1177_1049732320924625.pdf]

Glossary of Jefferson (2004) transcription conventions

tha**t**	Boldface indicates that a letter was produced in a crisp, dentalized way
underlining	Underlining indicates emphasis
[overlap]	Square brackets indicate overlapping talk
word=word	Equals sign indicates either that 1) a piece of talk was ‘latched’ onto another piece of talk or 2) two pieces of talk placed on separate lines of the transcript were, in the recording, produced without any break
::::	Colons indicate that the preceding sound was stretched
word.	Period indicates a falling intonation at the end of a unit of talk
word?	Question mark indicates rising intonation at the end of a unit of talk
word,	Comma indicates semi-rising, ‘continuing’ intonation at the end of a unit of talk
word__	Underscores indicate that there was no intonation change at the end of a unit of talk
(0.6)	Numbers in brackets indicate length of pauses, timed to the nearest tenth
word-	Dash indicates that the preceding sound was cut off
°word°	Degree signs indicate that the enclosed talk was noticeably quieter than surrounding talk
>word<	Inward-facing carets indicate that a word was noticeably faster than surrounding talk
.hhh/hhh	Indicates an in-breath (preceded by period) or outbreath (no period)

pt.	Indicates a lip smack
(word)	Single brackets indicate a best guess at talk that was unclear
((word))	Double brackets indicate information that was not part of the recording but could be useful for readers to know
